# Population-Based Outcomes in NSCLC

**DOI:** 10.1093/jncics/pkz022

**Published:** 2019-06-07

**Authors:** Martin J Edelman

**Affiliations:** Department of Hematology/Oncology, Fox Chase Cancer Center, Philadelphia, PA

A nihilistic approach to the treatment of non-small cell lung cancer (NSCLC) has characterized the predominant attitude of physicians as well as patients during most of my professional career. This attitude has persisted despite therapeutics that have demonstrated efficacy in terms both of quality and quantity of life since at least the early 1990s (1). The level of benefit and the trade-off in terms of toxicity has improved remarkably in the past 25 years. During this time, we have seen several key developments. The first has been the development of very effective antiemetic therapies with 5HT3 serotonin blockade, neurokinin 1 antagonists, and other agents that have made “traditional” platinum-based therapies more tolerable and have moved the site of treatment from the inpatient to the outpatient setting. Second, the reemergence of histology as a predictive marker for therapeutics (pemetrexed and bevacizumab) has resulted in a higher likelihood and duration of benefit from cytotoxic treatments. Third, the discovery of specific activating mutations responsive to tyrosine kinase inhibitors (interestingly, a result of clinical observation rather than laboratory discovery) and the widespread availability of genetic testing have allowed for the introduction of agents with the potential to benefit 80–90% of the patients undergoing therapy. Most recently, the introduction of immunotherapeutics has completely altered the landscape of therapy with the rapid introduction of four new agents in a variety of settings and combinations in NSCLC that substantially improve outcomes with acceptable levels of toxicity.

In this issue of the Journal, Maguire et al. provide a snapshot of clinical practice on a population basis just prior to the immunotherapy era in the California population (2). Although one can argue that the results are already obsolete with the rapid changes that have occurred since 2015, there are important aspects of this analysis that cannot be ignored. In terms of the results of treatment, the paper, for the most part, validates and extends prior work based on Surveillance, Epidemiology, and End Results (SEER)-Medicare data that was inherently restricted to a population age of older than 65 years (3). Most important, despite the substantial progress in the management of NSCLC that occurred prior to the immunotherapy era, a bare majority of patients with advanced NSCLC received the benefits of these advances. Although at least some of this can be attributed to patients presenting with severely impaired performance status and/or severe comorbidity, a depressingly large proportion of this gap between research and practice appears as the result of socioeconomics in the United States. However, the tendency toward undertreatment transcends national boundaries and health-care systems and has been seen in a similar study from Canada (4). Therefore, one needs to recognize that nihilistic views regarding treatment of this disease also play an important role in whether patients receive treatment.

An additional facet that needs to be considered is expertise. This is a somewhat taboo topic and runs counter to the goal of community-based care. However, there is consistent evidence that treatment in specialized centers is advantageous. Specifically, as demonstrated in the current paper as well as in other analyses, treatment in academic centers and more specifically, the National Cancer Institute–designated cancer centers, results in better outcomes (5). This is disappointing given the widespread availability and apparent use of guidelines (eg, by the American Society of Clinical Oncology or the National Comprehensive Cancer Network). Why does this occur? Partly a consequence of knowledge regarding the current standard of care but also a lack of the familiarity with complications and management of complications that can come only with experience.

Although most of the results of this analysis are consistent with the known benefits and outcomes of treatment of NSCLC, the lack of benefit from multiagent chemotherapy for squamous cell disease is unexpected. The data reported are insufficient to allow for firm conclusions and are in conflict with essentially all clinical trial data. Although squamous cell carcinoma patients may have an overall inferior prognosis, the relative benefits of cisplatin-based chemotherapy treatments are similar for squamous and nonsquamous subtypes (6–9). The result also conflicts with other population-based analyses from employing SEER-Medicare data, which did not identify differences between squamous and nonsquamous disease (3). Squamous cell carcinoma is a biologically distinct entity from the nonsquamous subtypes (which are themselves extraordinarily heterogeneous) (10). To date, no drug target has been validated in this disease despite numerous attempts, most notably through the Lung Master Protocol, SWOG S1400, a collaborative effort by the National Clinical Trials Network, industry, the Food and Drug Administration, and private organizations (Friends of the [National Institutes of Health] NIH) to find such drugs (11).

There are several areas of this report that require context and demonstrate the limitations of this type of analysis. One example is that the authors are critical of the relatively limited uptake of bevacizumab by oncologists despite a superior outcome in a randomized trial. It is important to recognize the bevacizumab-eligible population as an inherently better group of patients. The results of the control arm (carboplatin/paclitaxel) of the Eastern Cooperative Oncology Group (ECOG) 4599 trial (the study that led to the approval of bevacizumab for NSCLC) were markedly superior (overall survival 10.3 months vs 8.1 months) to a similarly treated arm on the prior ECOG trial (E1594), which did not have the restrictions on eligibility (no squamous carcinoma, anticoagulation, hemoptysis, central nervous system disease) (12,13). Furthermore, subsequent analyses of the ECOG trial indicated that there was no benefit from the addition of bevacizumab to chemotherapy in the elderly (defined as age >70 years) (14). These factors, coupled with the withdrawal of approval in metastatic breast cancer, likely accounts for the paucity of use of this drug (15).

Overall, the results of this study provide a snapshot of practice that confirms the results of clinical trials but demonstrates how socioeconomic features and physician attitudes strongly influence how these developments are applied. Overcoming these barriers is likely to remain an important challenge.

## Note

Affiliation of author: Department of Hematology/Oncology, Fox Chase Cancer Center, Philadelphia, PA.

## References

[1] Wakelee H, Kelly K, Edelman MJ. 50 years of progress in the systemic therapy of non-small cell lung cancer. Am Soc Clin Oncol Educ Book. 2014;34:177–189. 10.14694/EdBook_AM.2014.34.177 PMC5600272 24857075

[2] Maguire FB, Morris CR, Parikh-Patel A, et al First-line systemic treatments for stage IV non-small cell lung cancer in California: patterns of care and outcomes in a real-world setting. J Natl Cancer Inst. 2019. doi: 10.1093/jncics/pkz020. 10.1093/jncics/pkz020 PMC7050031 32328551

[3] Davidoff AJ, Tang M, Seal B, Edelman MJ. Chemotherapy and survival benefit in elderly patients with advanced non-small-cell lung cancer. J Clin Oncol. 2010;28(13):2191–2197. 20351329 10.1200/JCO.2009.25.4052

[4] Sacher AG, Le LW, Lau A, Earle CC, Leighl NB. Real-world chemotherapy treatment patterns in metastatic non-small cell lung cancer: are patients undertreated? Cancer. 2015;121(15):2562–2569. 25891153 10.1002/cncr.29386

[5] Ramalingam S, Dinan MA, Crawford J. Survival comparison in patients with stage IV lung cancer in academic versus community centers in the United States. J Thorac Oncol. 2018;13(12):1842–1850. 30312680 10.1016/j.jtho.2018.09.007

[6] Lilenbaum RC, Herndon JE II, List MA, et al Single-agent versus combination chemotherapy in advanced non-small-cell lung cancer: the Cancer and Leukemia Group B (study 9730). J Clin Oncol. 2005;23(1):190–196. 15625373 10.1200/JCO.2005.07.172

[7] Kelly K, Chansky K, Mack PC, et al Chemotherapy outcomes by histologic subtypes of non-small-cell lung cancer: analysis of the Southwest Oncology Group database for antimicrotubule-platinum therapy. Clin Lung Cancer. 2013;14(6):627–635. 23910067 10.1016/j.cllc.2013.06.010 PMC4122504

[8] Quoix E, Zalcman G, Oster JP, et al Carboplatin and weekly paclitaxel doublet chemotherapy compared with monotherapy in elderly patients with advanced non-small-cell lung cancer: IFCT-0501 randomised, phase 3 trial. Lancet. 2011;378(9796):1079–1088. 21831418 10.1016/S0140-6736(11)60780-0

[9] Feliciano JL, Le-Rademacher JG, Gajra A, et al Do older patients with non-small cell lung cancer also benefit from first-line platinum-based doublet chemotherapy? Observations from a pooled analysis of 730 prospectively-treated patients (Alliance Study A151622). J Geriatr Oncol. 2018;9(5):501–506. 29848457 10.1016/j.jgo.2018.05.006 PMC6233872

[10] Cancer Genome Atlas Research Network. Comprehensive genomic characterization of squamous cell lung cancers. Nature. 2012;489(7417):519–525. 22960745 10.1038/nature11404 PMC3466113

[11] Herbst RS, Gandara DR, Hirsch FR, et al Lung master protocol (Lung-MAP)—a biomarker-driven protocol for accelerating development of therapies for squamous cell lung cancer: SWOG S1400. Clin Cancer Res. 2015;21(7):1514–1524. 25680375 10.1158/1078-0432.CCR-13-3473 PMC4654466

[12] Sandler A, Gray R, Perry MC, et al Paclitaxel-carboplatin alone or with bevacizumab for non-small-cell lung cancer. N Engl J Med. 2006;355(24):2542–2550. 17167137 10.1056/NEJMoa061884

[13] Schiller JH, Harrington D, Belani CP, et al Comparison of four chemotherapy regimens for advanced non-small-cell lung cancer. N Engl J Med. 2002;346(2):92–98. 11784875 10.1056/NEJMoa011954

[14] Ramalingam SS, Dahlberg SE, Langer CJ, et al Outcomes for elderly, advanced-stage non small-cell lung cancer patients treated with bevacizumab in combination with carboplatin and paclitaxel: analysis of Eastern Cooperative Oncology Group Trial 4599. J Clin Oncol. 2008;26(1):60–65. 18165641 10.1200/JCO.2007.13.1144

[15] Vitry A, Nguyen T, Entwistle V, Roughead E. Regulatory withdrawal of medicines marketed with uncertain benefits: the bevacizumab case study. *J Pharm Policy Pract*. 2015;8:25. 10.1186/s40545-015-0046-2 PMC4610052 26483954

